# Systematic pore lipophilization to enhance the efficiency of an amine-based MOF catalyst in the solvent-free Knoevenagel reaction

**DOI:** 10.3762/bjoc.21.144

**Published:** 2025-09-09

**Authors:** Pricilla Matseketsa, Margret Kumbirayi Ruwimbo Pagare, Tendai Gadzikwa

**Affiliations:** 1 Department of Chemistry, Kansas State University, Manhattan, Kansas 66506, United States

**Keywords:** metal-organic frameworks, post-synthesis modification, supramolecular catalysis

## Abstract

We systematically lipophilized an amine-based metal-organic framework (MOF) catalyst and applied the functionalized MOFs to the Knoevenagel condensation reaction. A well-defined MOF material composed of both amine- and hydroxy-bearing linkers was reacted with a series of aliphatic isocyanates (isopropyl, *tert*-butyl, *n*-hexyl, and tetradecyl) and, incongruously, was found to preferentially react at the hydroxy groups. This selective functionalization yielded MOFs in which the catalytically active amines are confined within highly lipophilic pores, reminiscent of many enzyme active sites. We determined that systematically increasing the lipophilicity of the pores results in a commensurate increase of catalyst efficiency.

## Introduction

Most enzymatic reactions take place in multifunctional cavities in which multiple amino acid residues work cooperatively to orient and activate reactants [1–3]. These residues may also enhance covalent and/or acid–base catalysis via any combination of non-covalent interactions (hydrogen bonding, π–π stacking, lipophilic interactions, etc) [4–6]. Inspired by enzymes, Nature's most efficient catalysts, chemists have long endeavored to synthesize catalytic materials in which multiple functional groups are isolated together in confined space [7–9]. In the solid state, the generation of such multifunctional cavities has been pursued upon nanoporous scaffolds that include polymers of intrinsic microporosity (PIMs) [10–13], mesoporous silica materials (MSMs) [14–17], and metal-organic-frameworks (MOFs), among others [18–20]. Within this group of porous materials, MOFs boast the advantages of their crystallinity, the uniformity of their pores that are typically in the microporous range (5–20 Å), and the ability to fine-tune their pore chemical environment [21–22]. These attributes allow us to construct MOF-based catalysts with active sites that are isolated within cavities of the same size range as small molecules and whose walls are decorated with precisely located functional groups. We can rationally elaborate these functional groups to modulate catalytic performance and/or systematically investigate the influence of a particular chemical or structural property on catalyst efficiency [23].

**Figure 1:**
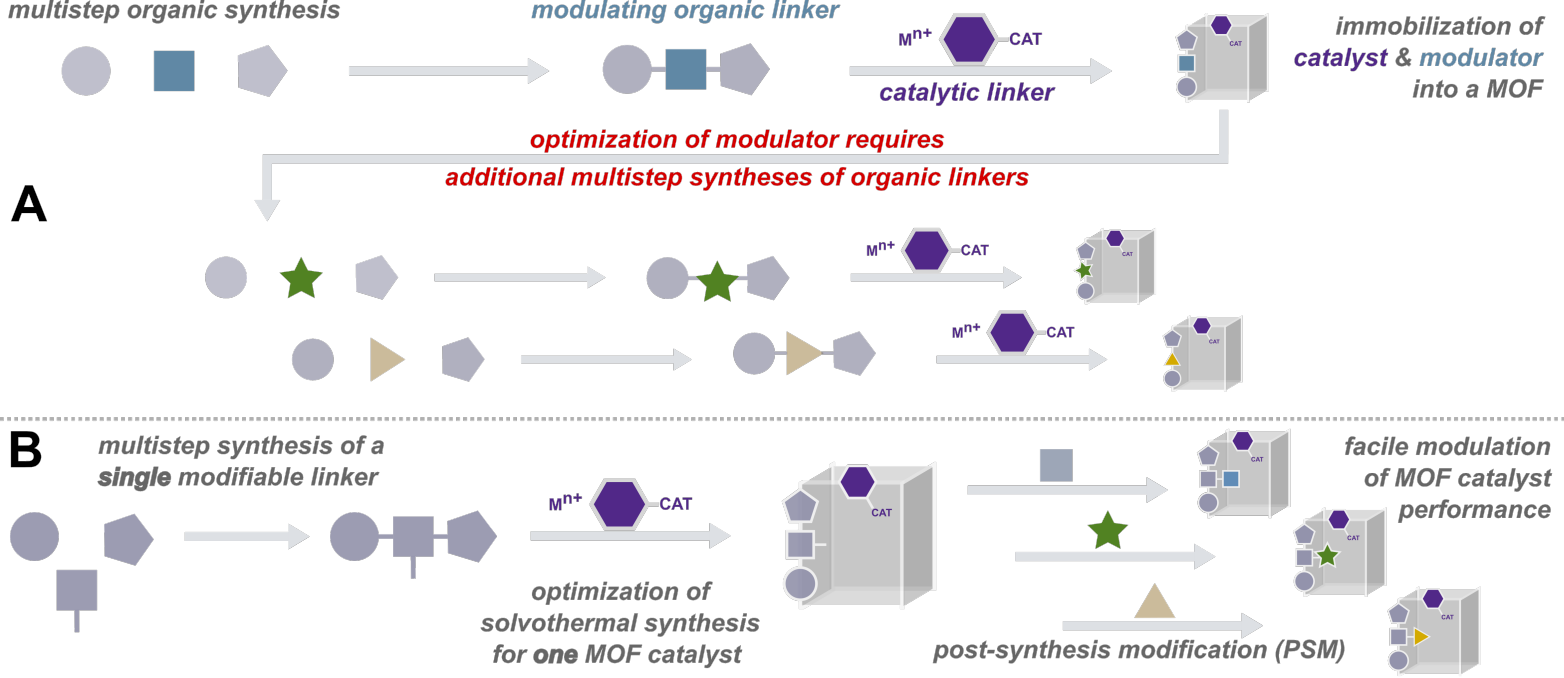
Schematic representation of the modulation of MOF pore environments. A) de novo synthesis of several MOFs requiring the multistep synthesis of different organic linkers. B) Synthesis of several frameworks from a single parent MOF using post-synthesis modification (PSM).

For examples of tailoring the pore environment in MOF-based catalysts to modulate catalytic performance, we can refer to the elegant work of Telfer and co-workers. In two separate reports, they synthesized well-defined MOFs composed of three different linkers: a proline-functionalized linker acted as the catalytic unit, while two auxiliary linkers were varied to alter catalyst activity and enantioselectivity [24], or product selectivity [25]. In those works, the researchers tailored their catalyst via de novo solvothermal synthesis of their MOFs using differently substituted auxiliary linkers; a non-trivial effort which involves the synthesis of several new organic linkers and their subsequent assembly into completely new frameworks (Figure 1A). In this report we describe, how a similar tailoring of a MOF’s pore environment, with consequent activity modulation, can be realized more efficiently using covalent post-synthetic modification (PSM) strategies [26–27]. Starting with a single MOF material that has both catalytic linkers and auxiliary linkers that bear reactive “tags”, we can graft additional functionalities onto the auxiliaries to adjust the steric and electronic environment of the catalytic units (Figure 1B).

The advantages of PSM as a strategy for generating MOF-based catalysts are that we can efficiently generate several MOF catalysts from a single parent framework. Additionally, we can introduce new functionality into a MOF without changing the framework topology, thus minimizing the number of variables to consider as we study the influence of a particular property on catalytic performance. Perhaps more importantly for enzyme-inspired materials, PSM allows us to incorporate functionalities that are pertinent to catalysis but that would normally interfere with MOF assembly, e.g. hydrogen bonding groups like –OH and –COOH that are difficult to obtain as free uncoordinated moieties within MOF pores [28–29]. Given these benefits, it is no surprise that PSM is a prevalent method for synthesizing MOF-based catalysts [30].

On this topic, our current work was inspired by Canivet et al. who previously reported the lipophilization of a MOF by grafting long alkyl chains to the *external* surfaces of its crystals. The active sites are believed to be coordinatively unsaturated zincs at the MOF surfaces, and their lipophilization resulted in a greater than ten-fold increase in the initial rate of the reaction [31]. The promotion of this reaction was attributed to the repulsion of the water by-product by the lipophilic surface, thereby preventing its interference with the Lewis acidic catalyst surface sites, but we wondered, if similar reaction acceleration of a condensation reaction could be achieved by the lipophilization of the *internal* surfaces of an amine-based MOF catalyst.

The majority of studies of lipophilic MOFs applied to catalysis have focused on lipophilization to prevent water-based catalyst decomposition, with only a few investigating how lipophilic pores surfaces can increase catalyst efficiency [32–33], despite enzymes employing such a strategy. The lipophilicity of enzyme active sites tends to improve reaction rates by increasing the binding affinity for the lipophilic reactants and by decreasing the energy required to desolvate acid/base amino acid catalysts [34–35]. Lipophilicity has also been found to be beneficial in condensation reactions as the removed water molecules are repelled by the hydrophobic environment, suppressing the hydrolysis reaction that would return the starting materials [36]. Thus, in this work, we investigate the influence of pore lipophilicity on the amine-catalyzed Knoevenagel condensation.

The Knoevenagel condensation reaction is a vital organic reaction involving the condensation of carbonyl compounds, such as aldehydes or ketones, with active methylene compounds [37]. The resulting α,β-unsaturated carbonyl products can then be further elaborated to form natural products, therapeutic agents, polymers, pesticides, and insecticides [38], which have important applications in the pharmaceutical and agrochemical industries [38–39]. Various types of catalysts are used to enhance the efficiency and selectivity of the Knoevenagel reaction, including Lewis acids, ureas/ thioureas, amino acids, and bases such as alkali metal hydroxides, alkali metal alkoxide, amines, etc. [37,40–44].

## Results and Discussion

**Figure 2:**
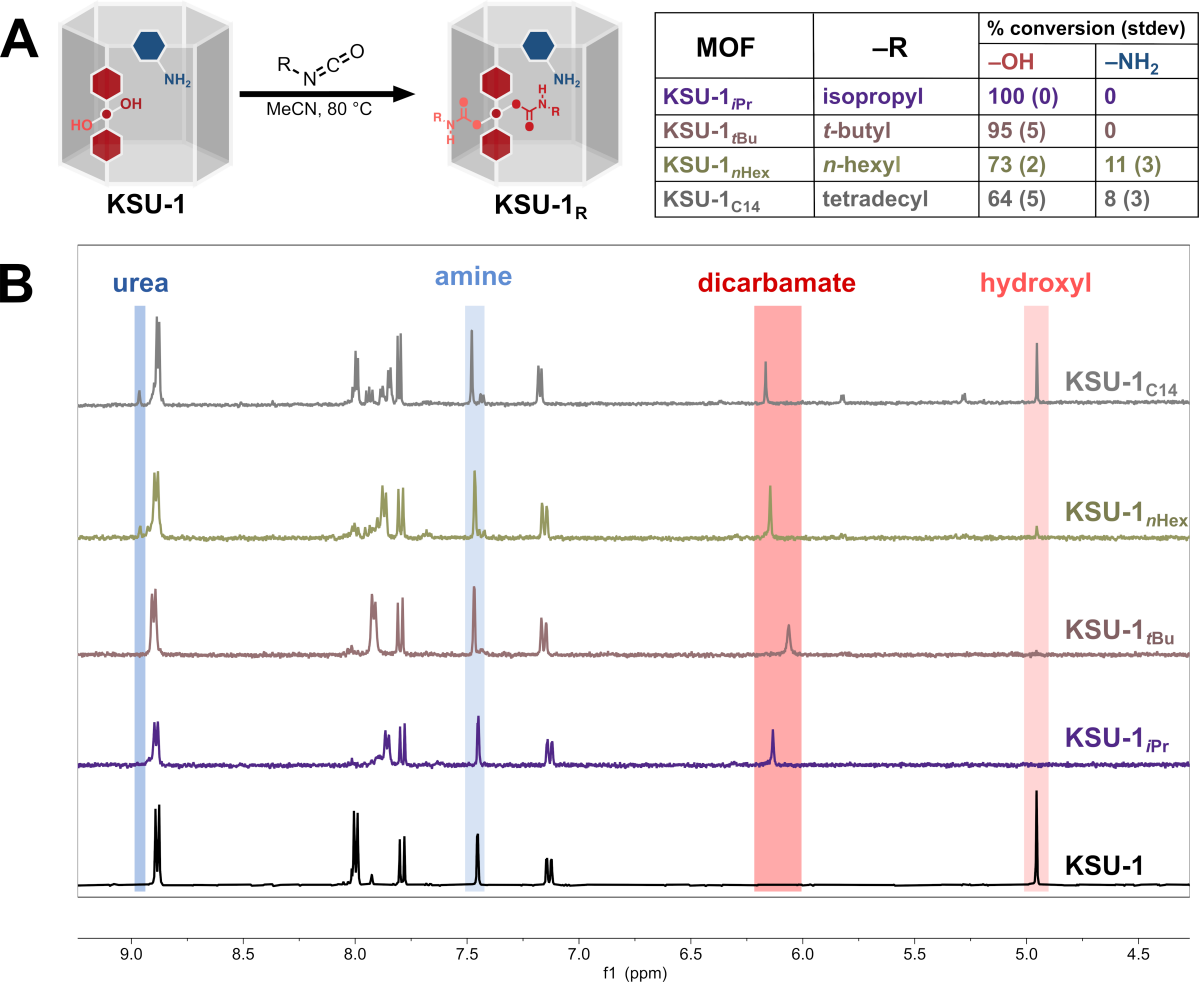
A) Schematic representation of the reaction of **KSU-1** with aliphatic isocyanates and the estimated conversions at –OH and –NH_2_. B) The corresponding ^1^H NMR spectra of the MOF reaction products digested in a solution of D_2_SO_4_ in DMSO-*d*_6_.

We opted for amine-based catalysis using our modifiable framework **KSU-1**, a pillared paddlewheel MOF assembled using zinc, 2-aminobenzene-1,4-dicarboxylic acid (H_2_BDC-NH_2_), and *meso*-α,β-di(4-pyridyl)glycol (DPG), as our parent MOF (Figure 2A). As this is a well-defined mixed-linker MOF with molecular formula Zn_2_(BDC-NH_2_)_2_(DPG), each unit cell has a 2:1 ratio of dicarboxylate to dipyridyl and, therefore, a 1:1 ratio of amine (–NH_2_) to hydroxy (–OH) groups. The Zn atoms in the paddlewheel metal clusters are coordinatively saturated [45], thus we anticipated that only the amine group would function as the catalytic unit for the Knoevenagel reaction, while the hydroxy group would serve as a handle through which we would tune the lipophilicity of the catalyst.

Recently, we found that isopropyl isocyanate reacts preferentially at the DPG hydroxy groups of **KSU-1** [46]; this, despite an earlier demonstration of the superior nucleophilicity of BDC-NH_2_ [45]. Interestingly, this apparent reversal in reactivity was most significant with aliphatic isocyanates, while the reactivity reverted to what is expected with the use of more activated isocyanates. Subsequently, we determined that, when incubated with secondary or tertiary isocyanates, **KSU-1** reacts exclusively at the hydroxy groups of the DPG linker before proceeding to react at the amines of BDC-NH_2_ (Table 1, entries 1 and 2). Thus, we had a method to generate, from a single framework, a series of amine-based MOFs whose pores are uniformly decorated with different lipophilic groups (Figure 2A). Using this strategy, we quantitatively functionalized the –OH groups of **KSU-1** with isopropyl and *tert*-butyl isocyanate. While primary isocyanates were less selective, starting to react at the amines before the hydroxy reaction was complete, we reacted **KSU-1** with *n*-hexyl and tetradecyl isocyanate as well (Table 1, entries 3 and 4) because we wanted to use longer alkyl chains to further increase the lipophilicity.

**Table 1:** Estimated conversions of the reactions of isocyanates with the –OH and –NH_2_ groups of **KSU-1** to form carbamates and ureas, respectively.^a^

Entry	Isocyanate	–OH % conv. (stdev)	–NH_2_ % conv. (stdev)

1	isopropyl^b^	100 (0)	0
2	*tert*-butyl^c^	94 (5)	0
3	*n*-hexyl^d^	73 (2)	11 (3)
4	tetradecyl^d^	64 (5)	8 (3)

^a^0.2 M in acetonitrile, 80 °C; ^b^3 h; ^c^4 h; ^d^2 h.

To obtain our bifunctional amine-based **KSU-1** MOF catalysts, we incubated **KSU-1** in a 0.2 M solution of the respective isocyanate in acetonitrile at 80 °C; **KSU-1** reacted with isopropyl, *tert*-butyl, *n*-hexyl, and tetradecyl isocyanate to generate **KSU-1_iPr_** and **KSU-1*_t-_*_Bu_**, **KSU-1*_n-_*_Hex_**, and **KSU-1_C14_**, respectively. Successful post synthetic reaction was observed by proton nuclear magnetic resonance (^1^H NMR) spectroscopy of the MOF product digested in D_2_SO_4_/DMSO-*d*_6_ (Figure 2B). Conversions were estimated by selecting the “same” proton in the starting material and in the products and integrating the corresponding peaks and setting the total to 100% (see Supporting Information File 1). We observed that the reactions with isopropyl isocyanate and *tert*-butyl isocyanate required 3 and 4 hours respectively to achieve complete conversion at the hydroxy group without any amine reaction. With *n*-hexyl isocyanate and tetradecyl isocyanate, reaction at the amine was observed after just one hour, before complete conversion at the hydroxy. To prevent excessive reaction at the amine, both reactions were stopped at 2 hours.

Aside from ^1^H NMR, the independent functionalization of **KSU-1** with isocyanates was also confirmed by conducting electrospray ionization mass spectrometry (ESI–MS) on samples of the MOF products digested by 1,4-diazabicyclo[2.2.2]octane (DABCO) (Figures S4–S7, Supporting Information File 1). For **KSU-1_iPr_** and **KSU-1*_t-_*_Bu_**, the mass spectra in negative mode had [M − H]^−^ peaks corresponding to deprotonated BDC-NH_2_, while in the positive mode, the [M + H]^+^ peaks indicated the presence of the protonated DPG dicarbamates, along with their various fragmentation products. The mass spectra for **KSU-1*_n-_*_Hex_** and **KSU-1_C14_**, show evidence of urea products in negative mode, and protonated DPG carbamates along with their fragmentation products in positive mode (Figures S4–S7, Supporting Information File 1). Powder X-ray diffraction (PXRD) confirmed that crystallinity was preserved even after complete functionalization of DPG (Figure S8, Supporting Information File 1).

**Scheme 1:**
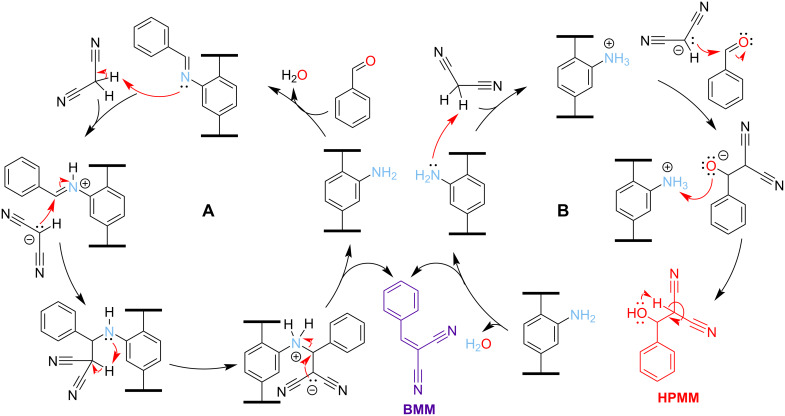
Probable mechanisms for the Knoevenegel condensation reaction between benzaldehyde and malononitrile catalyzed by a MOF-immobilized amine to form benzylidenemalononitrile (**BMN**). A) Mechanism in which the amine catalyst first undergoes imine condensation with benzaldehyde. B) Mechanism in which the amine acts as a base, deprotonating malononitrile.

To test the catalytic behavior of our amine-based lipophilic MOF catalysts, we chose the Knoevenagel condensation between benzaldehyde and malononitrile to form benzylidenemalononitrile (**BMN**). In a typical primary amine-catalyzed Knoevenagel condensation, the amine would undergo imine condensation with benzaldehyde. The imine would then deprotonate malononitrile, and the resulting carbanion would react with the imine, releasing the final product and the amine catalyst (Scheme 1A) [47–48]. However, there is another possible mechanism where malononitrile is deprotonated by the amine catalyst. Here, the resulting carbanion would attack benzaldehyde to form 2-(hydroxy(phenyl)methyl)malononitrile (**HPMM**) as an intermediate that then loses a water molecule, yielding **BMN** as the final product (Scheme 1B) [49].

**Figure 3:**
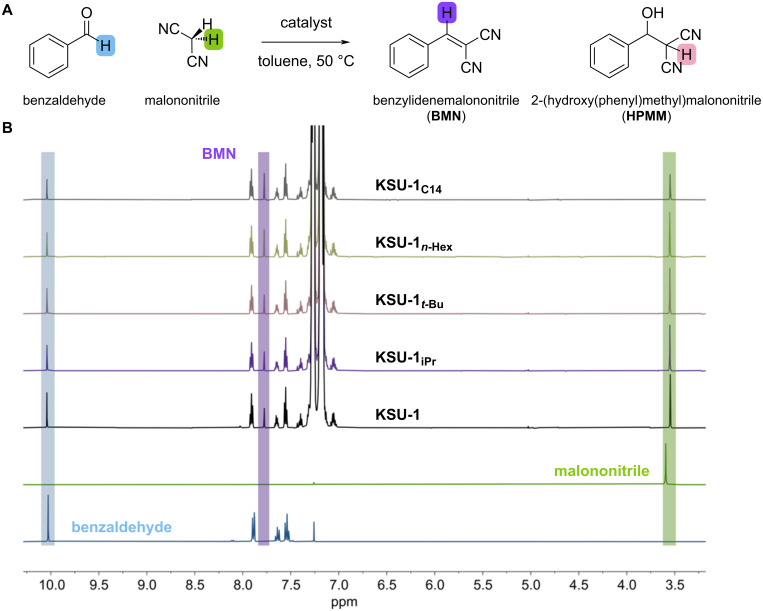
A) Schematic representation of the reaction between benzaldehyde and malononitrile to form benzylidenemalononitrile (**BMN**). B) Representative ^1^H NMR spectra for the reaction of benzaldehyde and malononitrile in toluene with dodecane as internal standard, analyzed after 30 minutes.

In the initial trial, 12 mol % of the MOF catalyst was added to a vial containing benzaldehyde, malononitrile, toluene as solvent, and dodecane as internal standard, and the reaction was shaken at 50 °C. Aliquots were collected at 30 minutes, diluted in CDCl_3_ and conversions were determined by analyzing the ^1^H NMR specta of the samples (Figure 3). The results showed a general increase in catalytic efficiency as we increased the lipophilicity of the MOF pores. However, the observed differences in conversion between the lipophilicized catalysts was marginal, with a variation of ≈8% (Table 2A). We were concerned that dodecane, our aliphatic internal standard, was negatively affecting the influence of lipophilization. Our working hypothesis was that increasing the lipophilicity of the MOF promotes the exclusion of water from the pores, which increases the rate of the condensation reaction. Thus, we initially supposed that, though dodecane was only 5.6 vol % to toluene, its addition was decreasing the difference in lipophilicity between the MOF and the bulk solution, reducing the driving force to remove water from the MOF pores.

**Table 2:** Comparison of Knoevenagel catalysis results under different conditions.^a^

Entry	Catalyst	% Conversion			

		**A.** toluene + dodecane^b^	**B.** toluene^c^	**C.** toluene^d^	**D.** neat^e^

1	none^f^	0 (0)	0 (0)	0 (0)	3.5 (1)
2	**KSU-1**	36 (2)	8 (1)	3 (0)	43 (2)
3	**KSU-1** **_iPr_**	48 (1)	19 (2)	3 (0.5)	56 (3)
4	**KSU-1** ***_t-_*** **_Bu_**	51 (1)	23 (4)	2.5 (0.5)	65 (5)
5	**KSU-1** ***_n-_*** **_Hex_**	51 (1)	15 (3)	5 (1)	67 (5)
6	**KSU-1** **_C14_**	56 (3)	16 (5)	8 (0)	77 (5)

^a^0.0625 mmol benzaldehyde, 0.068 mmol malononitrile, 50 °C, 30 min; ^b^12 mol % catalyst, 0.083 mmol dodecane, 250 μL toluene; ^c^12 mol % catalyst, 250 μL toluene; ^d^1.5 mol % catalyst, 250 μL toluene; ^e^1.5 mol % catalyst; ^f^0 mol % catalyst in all cases.

When we compared the conversions we obtained using the dodecane calibration curve with those determined by directly comparing the integrations of the benzaldehyde and **BMN** protons in the ^1^H NMR spectra, we found little difference (Table S2, Supporting Information File 1). Thus, we decided to discontinue the addition of internal standard to the catalysis reactions. Interestingly, performing the reaction without dodecane revealed significantly lower conversions (Table 2B). This led us to a modified hypothesis; namely that in a lipophilic environment, such as a lipophilicized pore or one containing a hydrophobic solvent, water molecules prefer to self-assemble to minimize interactions with the nonpolar solvent or pore walls. These clusters could be in the MOF channels or could conceivably be driven out of the hydrophobic frameworks into the bulk solvent where they can agglomerate with other water clusters to reduce overall surface tension.

**Figure 4:**
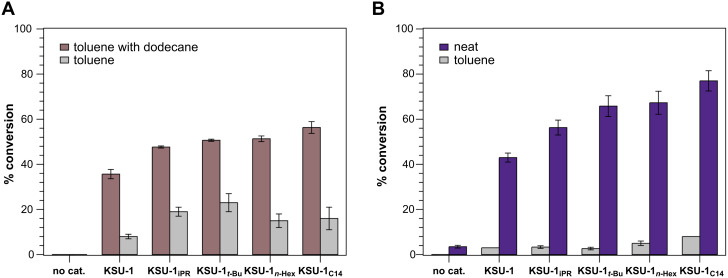
Graphical representation of the Knoevenagel catalysis results. A) Comparison of the reaction in toluene with and without internal standard using 12 mol % catalyst. B) Catalysis performed under neat conditions and in toluene using 1.5 mol % catalyst.

Along with reduced conversions, we also found that our anticipated trend in catalyst efficiency, i.e. increasing conversions with the increasing lipophilicity of the carbamate substituents, was not consistently followed (Table 2B). While conversions increased going from no substitution, to isopropyl, then *tert*-butyl, subsequent increases in lipophilicity with *n*-hexyl and tetradecyl resulted in decreased conversions; a trend that can be more clearly perceived in Figure 4A. The combined dodecane-free results led us to speculate that it would be better to remove solvent as a variable and rely solely on MOF modification to create a lipophilic environment in the pores. However, because solvents improve the solubility of the reactants and products, making it easier for the reagents to reach the active sites, we were concerned that performing the reaction under neat conditions would be detrimental: malononitrile is a solid and the rapid formation of solid product **BMN** often results in a thick sludge that hinders the flow of the reaction solution. Additionally, we were concerned that the lack of solvent, would mean that the MOF catalyst would not be completely submerged in the reaction mixture.

To address both concerns of performing the neat Knoevenagel reaction – product precipitation and the incomplete submersion of the MOF catalyst – we reduced the catalyst amount to 1.5 mol %. Surprisingly, the reaction conversions were higher than those in toluene with 12 mol % catalyst, and significantly so (ca. 10-fold) with a more appropriate comparison at the same catalyst loading of 1.5 mol% (Table 2C and D). Further, to our delight, **KSU-1_C14_** was the most active catalyst achieving 77% conversion vs 43% for **KSU-1** after 30 min. In addition, a remarkably clear and gradual increase in the rate of reaction was observed from **KSU-1** < **KSU-1_iPr_** < **KSU-1*_t-_*_Bu_** < **KSU-1*_n-_*_Hex_** < **KSU-1_C14_** (Figure 4B) even though roughly 10% of the –NH_2_ groups in both **KSU-1*_n-_*_Hex_** and **KSU-1_C14_** had been converted to the less catalytically active alkyl ureas [50].

We should also point out that, despite the lower extent of alkyl grafting, the –CH_2_– content in the pores of **KSU-1*_n-_*_Hex_** and **KSU-1_C14_** is still greater than for **KSU-1_iPr_** and **KSU-1*_t-_*_Bu_**, assuming a uniform distribution of alkyl chains. Additionally, although the introduction of large alkyl substituents in a MOF is associated with a reduction in pore accessibility, the lower percentage of alkyl grafting for both **KSU-1*_n-_*_Hex_** and **KSU-1_C14_** results in similar solvent accessible volumes for all the modified MOFs, as shown by thermogravimetric analysis (TGA; Figure S9 in Supporting Information File 1). Thus, our results indicate that catalytic efficiency improves with the increasing lipophilicity of the alkyl chains, which is a result of the increase in surface area of the alkyl groups [51].

Of further interest are the results of the catalysis under the same neat conditions using the dimethyl ester of our catalytically active linker, Me_2_-BDC-NH_2_ (Table S3, Supporting Information File 1). We found that the conversions were significantly better than those reported for H_2_BDC-NH_2_ under more forcing conditions (60 °C, 6 h) [52], which makes sense given that the methyl substituents on the carboxylate increase the basicity of –NH_2_, which should increase the catalytic capability. What is surprising, however, is that the conversions for Me_2_BDC-NH_2_ are much lower than those observed for all our **KSU-1** derivatives despite the presumably lower basicity of the MOF-immobilized amino groups due to the dicarboxylates coordinating to Zn^2+^ clusters. This unexpected result indicates that the reaction experiences a positive confinement effect which is enhanced further by increasing the lipophilicity of the pore environment (Figure S3, Supporting Information File 1).

**Figure 5:**
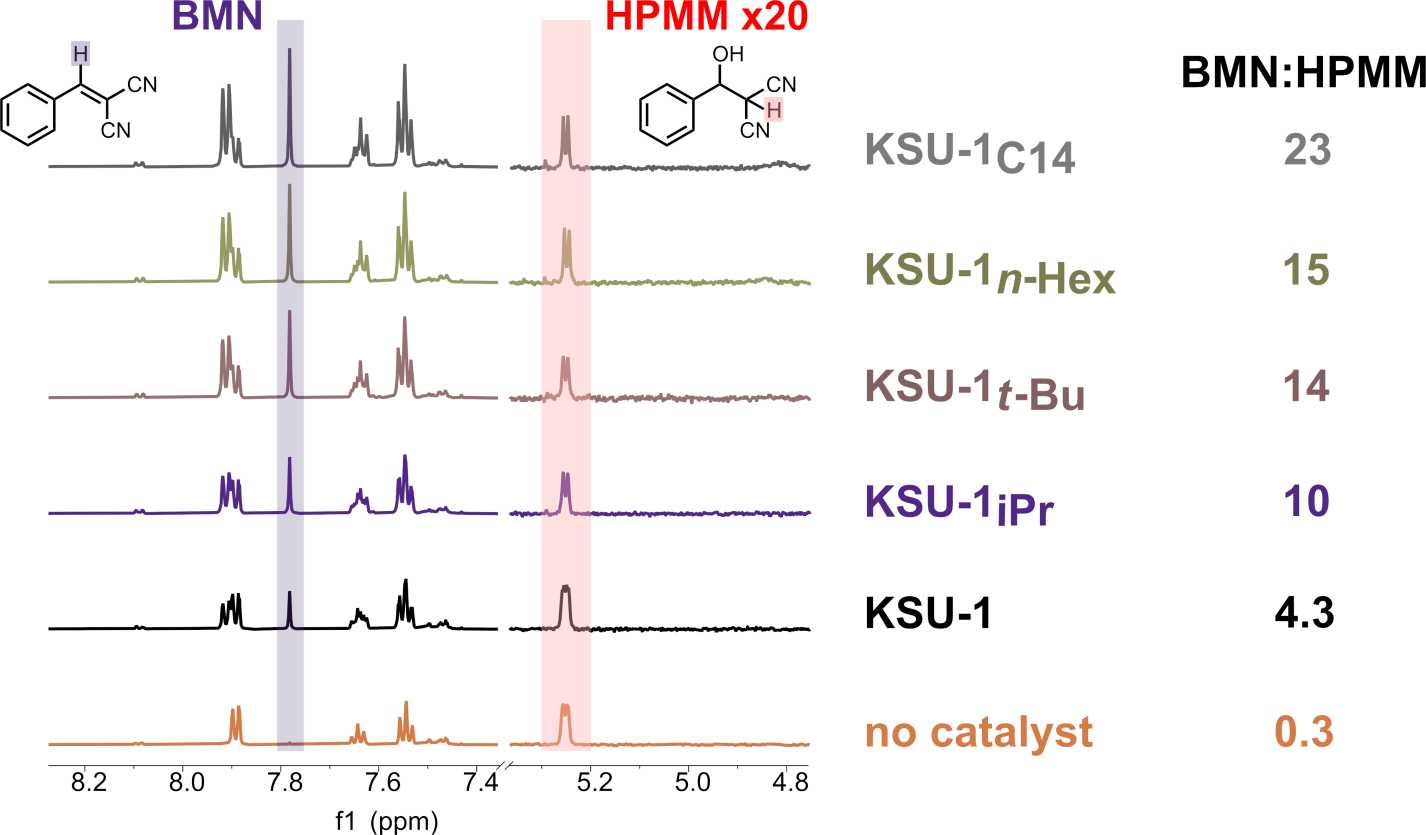
Left: comparison of **BMN** and **HPMM** protons in ^1^H NMR spectra. Note that the peaks corresponding to **HPMM** are under ×20 magnification. Right: ratios of **BMN**:**HPMM** products for each catalyst.

Finally, we emphasize that our study was not geared toward obtaining particular conversions with our catalysts, but rather at investigating the effect that systematic pore environment modulation has on catalyst reactivity; specifically, how lipophilization affects a condensation process like the Knoevenagel reaction. As such, it was also interesting to observe differences in the relative amount of reaction intermediate depending on the lipophilicity of the pores. Under solvent-free conditions, we observed the formation of 2-(hydroxy(phenyl)methyl)malononitrile (**HPMM**, Figure 5), an intermediate which is subsequently dehydrated to yield the main product (**BMN**) as the reaction progresses [53]. Looking at the ratio of final product to intermediate (**BMN**:**HPMM**), we observed that relatively more of the hydroxy intermediate was observed with the unfunctionalized KSU-1 catalyst (Figure 5) when compared to the lipophilicized catalysts, with the amount of **HPMM** decreasing with increasing aliphatic chain surface area. This trend suggests that the lipophilic surfaces may destabilize hydrophilic intermediates, promoting faster conversion of **HPMM** to **BMN**, similar to the ground-state destabilization of polar substrates observed in enzymes with lipophilic pockets [54–55]. It should be noted that this result is in contrast to a previous reportthat observed an *increase* in the intermediate when a non-polar solvent is used in the grinding-assisted Knoevenagel reaction of the same reactants [56].

## Conclusion

By employing a covalent post synthesis modification strategy that selectively introduces lipophilic functionality into MOFs, we confined catalytically active amines within MOF pores of systematically increasing lipophilicity. Our results reveal a clear correlation between increased pore lipophilicity and enhanced catalytic activity. Additionally, increasing lipophilicity resulted in congruent changes in the distribution of intermediate versus product during the reaction. Both these behaviors, increased efficiency and different intermediate:product distributions, call to mind the effect of lipophilic pockets in enzyme catalysis, and they offer a view to the possibilities that can be achieved in enzyme-inspired catalysis via the rational functionalization of MOF pores.

## Supporting Information

File 1 Experimental and characterization data.

## Data Availability

Data generated and analyzed during this study is available from the corresponding author upon reasonable request.
