# Supplementary material for: Systematic pore lipophilization to enhance the efficiency of an amine-based MOF catalyst in the solvent-free Knoevenagel reaction
Source: Beilstein J Org Chem. 2025 Sep 9;21:1854–63. doi: 10.3762/bjoc.21.144 (PMC12434926; doi:10.3762/bjoc.21.144)
Supplement: File 1 — Experimental and characterization data. [file Beilstein_J_Org_Chem-21-1854-s001.pdf]

BEILSTEIN JOURNAL OF ORGANIC CHEMISTRY

Supporting Information

for

Systematic pore lipophilization to enhance the efficiency of an amine-based MOF catalyst in the solvent-free Knoevenagel reaction

Pricilla Matseketsa, Margret Kumbirayi Ruwimbo Pagare and Tendai Gadzikwa

Beilstein J. Org. Chem. **2025,** *21,* 1854–1863. doi:10.3762/bjoc.21.144

Experimental and characterization data

License and Terms: This is a supporting information file under the terms of the Creative Commons Attribution License (https://creativecommons.org/licenses/by/4.0). Please note that the reuse, redistribution and reproduction in particular requires that the author(s) and source are credited and that individual graphics may be subject to special legal provisions.
The license is subject to the *Beilstein Journal of Organic Chemistry* terms and conditions: (https://www.beilstein-journals.org/bjoc/terms)

Table of contents

Materials S2

Synthetic procedures S2

Proton nuclear magnetic resonance (^{1}H NMR) S3

High-resolution mass spectrometry (HRMS) S4

Powder X-ray diffraction (PXRD) S10

Thermogravimetric analysis (TGA) S10

MATERIALS

All chemicals were used as received from commercial sources unless otherwise noted. Isopropyl isocyanate (98%), *tert*-butyl isocyanate (97%), tetradecyl isocyanate (97%) were purchased from Sigma Aldrich. Hexyl isocyanate (99%), 2-aminoterephthalic acid ($BDC-NH_2$, 99%), and dimethyl aminoterephthalate ($Me_2-BDC-NH_2$, 99%) were purchased from Thermo Scientific. Meso-α,β-di(4-pyridyl)glycol (>98%) (DPG) and malononitrile (97%) were purchased from TCI. Sulfuric acid-d_2 (D_2SO_4, 98 wt % in D_2O, 99.5+% atom D) was purchased from Acros Organics. *N*,*N*-Dimethylformamide (DMF), toluene, acetonitrile and benzaldehyde were purchased from Fisher Scientific (ACS certified). Zinc nitrate hexahydrate from Strem Chemicals and 1,4-diazabicyclo[2.2.2]octane (DABCO) (95%) was purchased from Oakwood Products. Dimethyl sulfoxide-d_6 (DMSO-d_6, 99.9 atom % D) was purchased from Cambridge Isotopes Laboratories.

SYNTHETIC PROCEDURES

KSU-1 was synthesized according to the literature procedure.[1]

Syntheses of KSU-1$_R$: General procedure for the reaction of KSU-1 with isocyanates.

In a typical experiment, ≈10 mg of **KSU-1** (0.012 mmol) (–OH (0.025 mmol) and $–NH_2$ (0.025 mmol)) as-synthesized in DMF was transferred to a 1-dram vial. Then, 2 mL of a 0.2 M solution of the isocyanate (0.4 mmol) in acetonitrile were added to the vial. The reaction was left at 80 °C with continuous mixing on a Corning LSE Low Speed Orbital Shaker. Samples of the reacted MOF were taken at 3 h, washed in acetonitrile 3 times filtered, and digested for analysis.

General procedure for catalysis

A suspension of the MOF catalyst in the reaction mixture was allowed to react at 50 °C with continuous mixing on a Corning LSE Low Speed Orbital Shaker for 30 min. After quenching by cooling to room temperature, $CHCl_3$ was added to separate the product from the solid catalyst. The supernatant was analyzed by ^1H NMR and the yield determined by comparing the integration of the signals corresponding to benzaldehyde to those corresponding to the products. Otherwise, the benzaldehyde/dodecane calibration curve was used (see below).

Toluene + dodecane

MOF material (0.0075 mmol; 12 mol %) was introduced into a 0.10 mL glass vial. Benzaldehyde (6.35 µL; 0.0625 mmol; 0.25 M) and malononitrile (4.5 mg; 0.068 mmol; 0.272 M) were then added. Toluene (250 µL) was added and the vial was sealed.

Toluene

MOF material or $Me_2-BDC-NH_2$ (0.0075 mmol; 12 mol %) was introduced into a 0.10 mL glass vial. Benzaldehyde (6.35 µL; 0.0625 mmol; 0.25 M) and malononitrile (4.5 mg; 0.068 mmol; 0.272 M) were then added. Toluene (250 µL) and dodecane (14.2 µL; 0.0625 mmol; 0.25 M) were added and the vial was sealed.

Neat

MOF material or $Me_2-BDC-NH_2$ (0.0075 mmol; 1.5 mol %) was introduced into a 0.10 mL glass vial. Benzaldehyde (0.5 mmol; 50.8 µL) and malononitrile (0.55 mmol; 36.5 mg) were then added and the vial was sealed.

1. Cohen, S. M. *Chem. Rev.* **2012,** *112,* 970–1000. doi:10.1021/cr200179u

PROTON NUCLEAR MAGNETIC RESONANCE (^{1}H NMR)

Spectra were recorded on a Bruker Avance NEO spectrometer (400 MHz for ^{1}H, Bruker BioSpin, Billerica, MA, USA). NMR chemical shifts are reported in ppm against a residual solvent resonance as the internal standard (δ(DMSO-d_6) = 2.5 ppm). In a typical analysis, MOF materials stored in MeCN were isolated by vacuum filtration and then evacuated in a vacuum oven at 80 °C overnight. Evacuated MOF samples (5–6 mg) were transferred into an NMR tube and DMSO-d_6 (0.55 mL) was added. Subsequently, D_2SO_4 (0.09 mL, 98 wt % in D_2O) was also added. The tubes were capped and sonicated until all the solid was dissolved (≈1 min).

Post-Synthesis Modification Conversion Estimation

Integration areas of peaks corresponding to the “same” proton in the product and starting material were compared (see Figure S1 for examples).

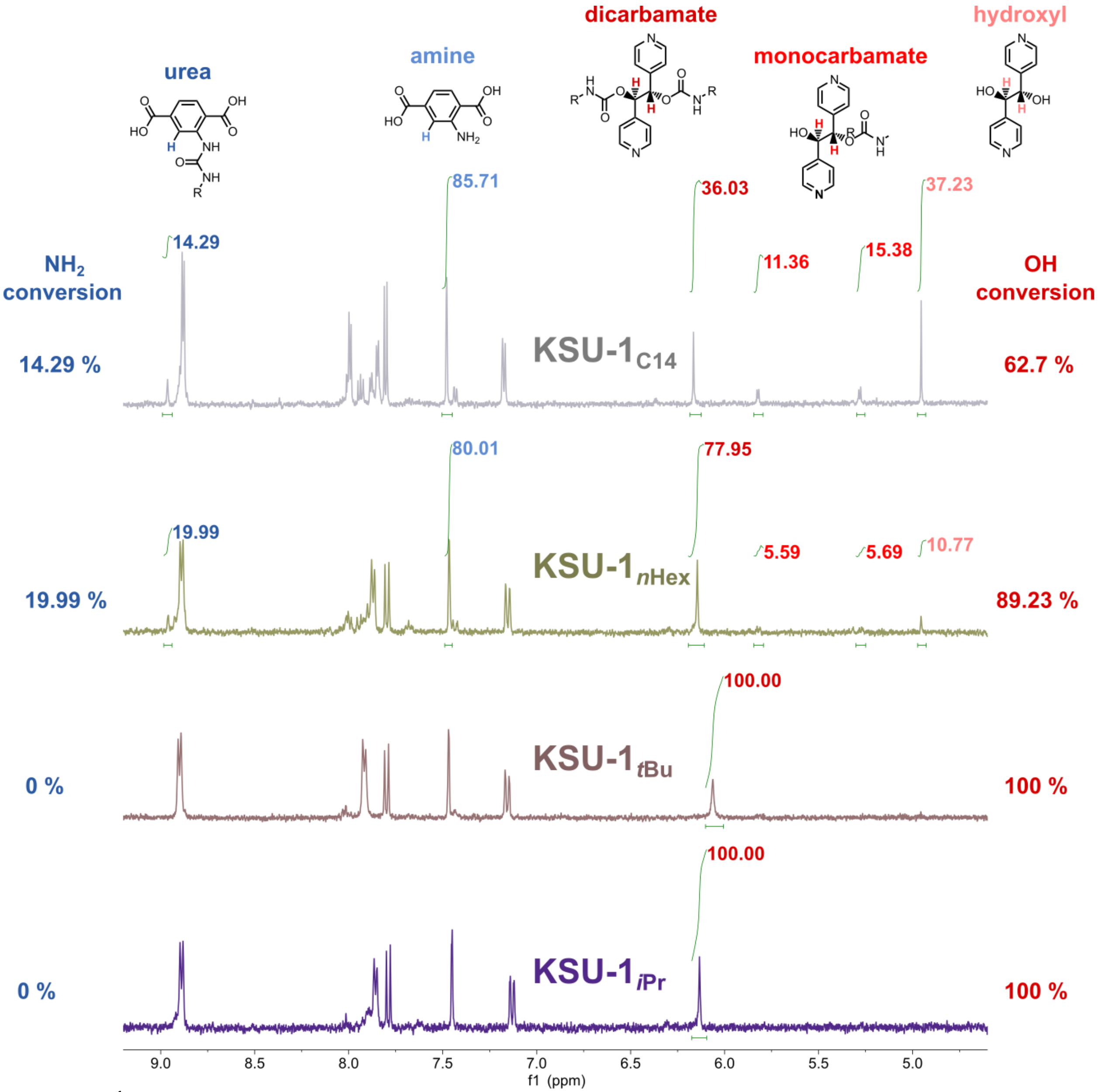

Figure S1: ^{1}H NMR of the products of the reaction of **KSU-1** with aliphatic isocyanates to form **KSU-1$_R$** with their respective integrations used to calculate conversions.

Dodecane calibration curve

Benzaldehyde (0.5 mmol; 50.8 µL) and dodecane (0.5 mmol; 113.6 µL) were added into two separate vials followed by 1.00 mL of toluene into each vial to make 0.50 M solutions. These solutions and toluene were added to NMR tubes to form samples A–E. To each sample, 550 µL of $CDCl_3$ was added and they were analyzed by ^1H NMR. The ratios of the areas of the benzaldehyde signal at 10.03 ppm (–C*H*O, 1H) vs the dodecane signal at 0.89 ppm (–CH_3, 6H) were determined for each sample and plotted vs % benzaldehyde.

Table S1: Table of composition of calibration curve samples.

	A	B	C	D	E
benzaldehyde µL	125	100	75	50	25
dodecane 0.50 M/µL	125	125	125	125	125
toluene/µL	0	25	50	75	100
[benzaldehyde]	0.25 M	0.20 M	0.15 M	0.10 M	0.05 M
[dodecane]	0.25 M	0.25 M	0.25 M	0.25 M	0.25 M
mol % benzaldehyde/dodecane	100	80	60	40	20

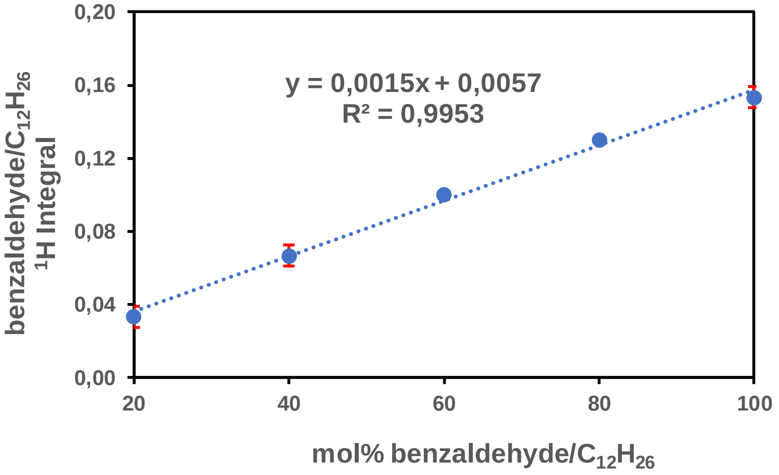

Figure S2: Calibration curve of the benzalydehyde proton with dodecane internal standard.

Determining benzaldehyde conversion

Internal standard method: The ratios of the areas of the benzaldehyde signal at 10.03 ppm vs the dodecane signal at 0.89 ppm were determined and plugged into the calibration curve to determine the mol % of benzaldehyde vs dodecane.

No internal standard method: The areas of the benzaldehyde signal at 10.03 ppm vs the benzylidenemalononitrile (BMN) signal at 7.78 ppm were summed and set to 100. The area of the peak corresponding to BMN was taken as the % conversion.

Table S2: Comparison of conversions obtained comparing the integral of the benzylidenemalononitrile proton to that of the dodecane internal standard (I.S.) vs comparing to the benzaldehyde (BA) proton.[a]

Entry	Catalyst	% Conversion					
		30 min. (I.S.)	30 min. (BA)	3 h (I.S.)	3 h (BA)	6 h (I.S.)	6 h (BA)
1	no catalyst	0	0	0	0	0	0
2	**KSU-1**	37	37	75	77	90	97
3	**KSU-1$_{iPr}$**	37	37	87	90	96	97
4	**KSU-1$_{t\text{-}Bu}$**	42	44	90	90	96	100
5	**KSU-1$_{n\text{-}Hex}$**	54	57	89	90	96	97
6	**KSU-1$_{C14}$**	58	64	92	90	97	100

[a]0.0625 mmol benzaldehyde, 0.068 mmol malononitrile, 0.083 mmol dodecane, 250 µL toluene,12 mol % cat., 50 °C.

Table S3: Conversions and BMN/HPMM ratios for all catalysis under neat conditions.[a]

Entry	Catalyst	% Conversion (stdev)	BMN/HPMM (stdev)
1[b]	-	3.5 (1)	0.3 (0.1)
2	**Me_2-BDC-NH_2**	15 (2)	1 (0.2)
3	**KSU-1**	43 (2)	4 (0.4)
4	**KSU-1$_{iPr}$**	56 (3)	10 (2)
5	**KSU-1$_{t\text{-}Bu}$**	65 (5)	14 (3)
6	**KSU-1$_{n\text{-}Hex}$**	67 (5)	15 (4)
7	**KSU-1$_{C14}$**	77 (5)	23 (7)

[a]0.0625 mmol benzaldehyde, 0.068 mmol malononitrile, 1.5 mol % cat., 50 °C. [b]0 mol % cat.

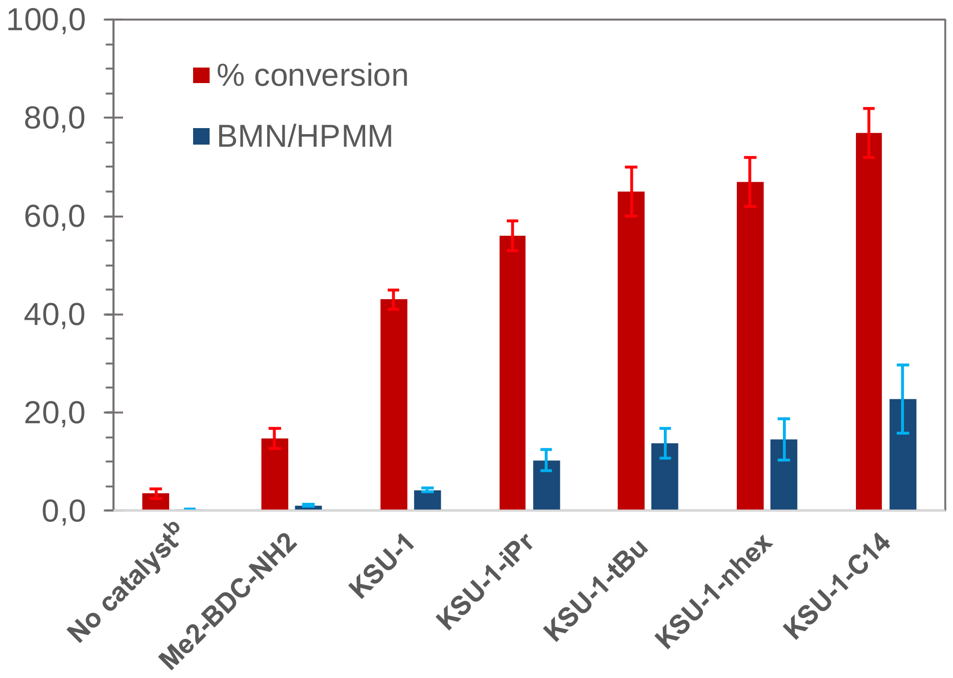

Figure S3: Plot showing conversions and BMN/HPMM ratios for all catalysis under neat conditions. 0.0625 mmol benzaldehyde, 0.068 mmol malononitrile, 1.5 mol % cat., 50 °C. [b]0 mol % cat.

HIGH-RESOLUTION MASS SPECTROMETRY (HRMS)

MOF samples (≈5 mg) were placed in a solution of DABCO (10 mg) and 0.25 mL of DMSO in a 2-dram vial. The vial was sonicated for ≈1 min then heated at 80 °C overnight. A small amount of residue was filtered out and the resulting solution was analyzed using a Xevo G2-XS QTof quadrupole time-of-flight mass spectrometer coupled with an ACQUITY M-class UPLC and a NanoLockSpray dual electrospray ion source. Mass spectra were acquired in "eXtreme Resolution" mode.

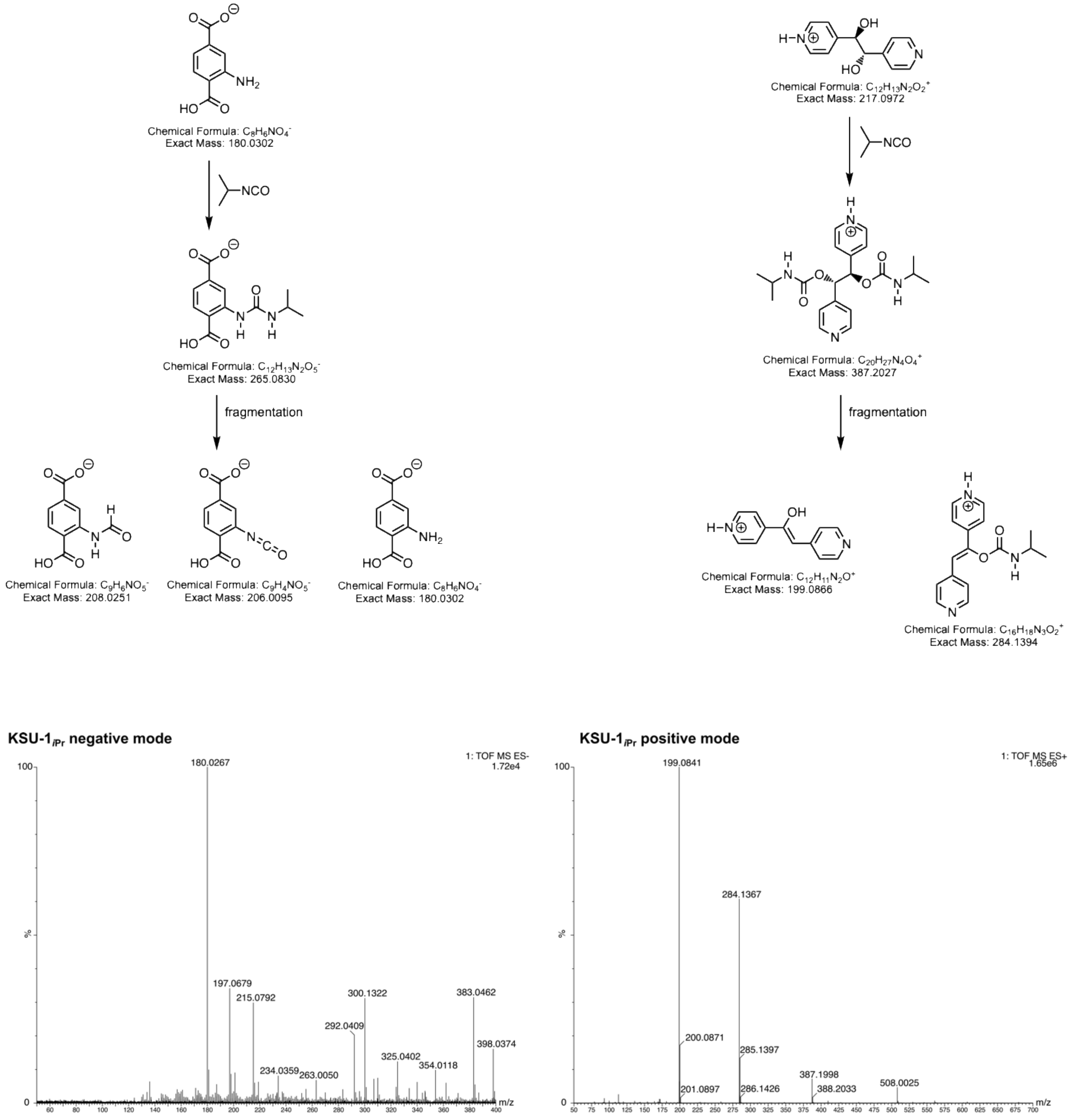

Figure S4: HRMS of **KSU-1** after reacting with isopropyl isocyanate to form **KSU-1$_{\mathbf{iPr}}$**. Left: the negative mode has no indication of the BDC urea product (*m*/*z* = 265.0267), though the [BDC-NH_2-H^+] starting material peak could also be a fragmentation product. Right: the positive mode has *m*/*z* peaks corresponding to [$DPG_{dicarbamate}$+H^+] (*m*/*z* = 284.1394) and its various fragmentation products.

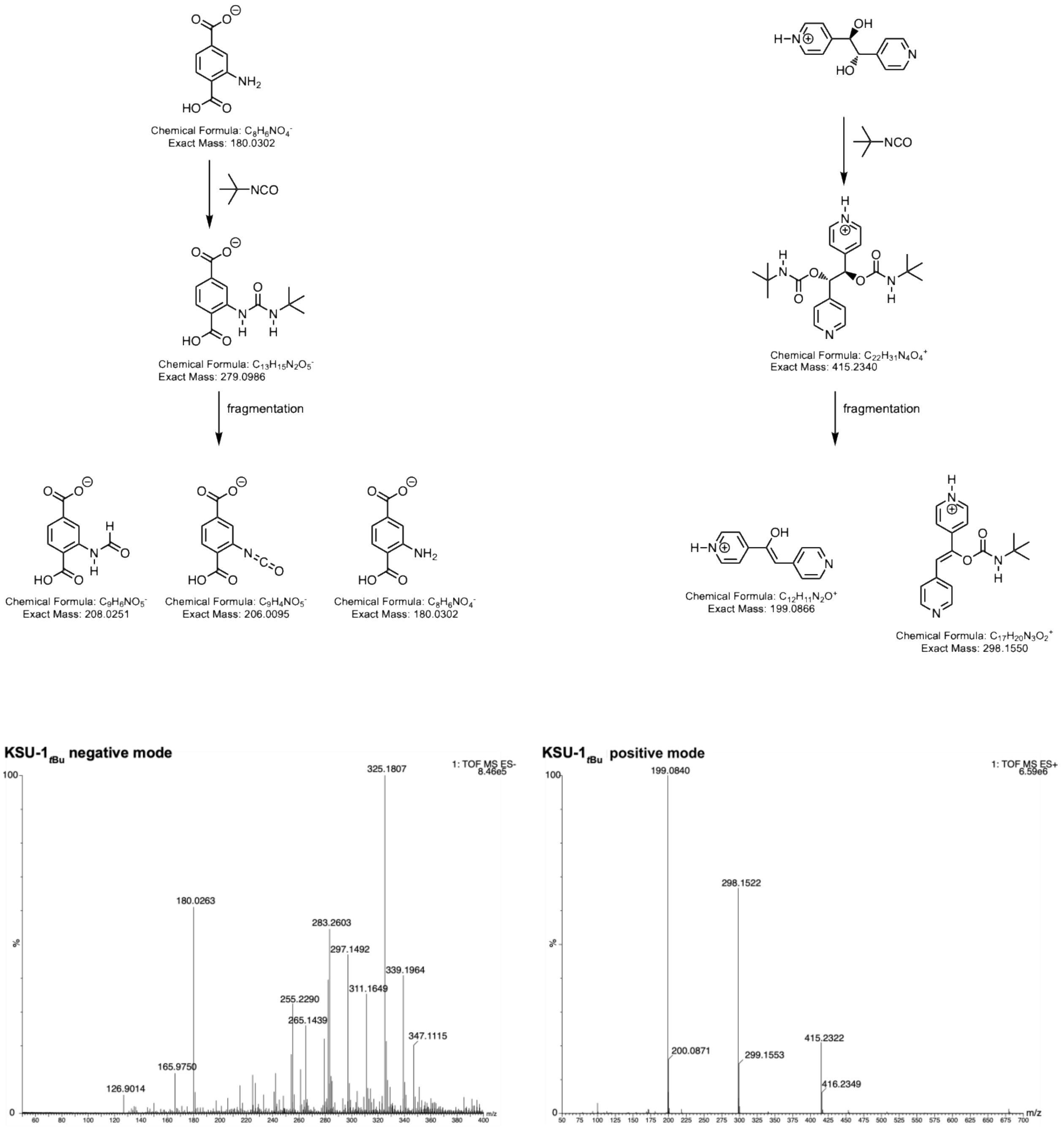

Figure S5: HRMS of **KSU-1** after reacting with *tert*-butyl isocyanate to form **KSU-1$_{t\text{-Bu}}$**. Left: the negative mode has no indication of the BDC urea product (*m*/*z* = 279.0986), though the [BDC-NH_2-H^+] starting material peak could also be a fragmentation product (note the presence of an unidentified mass at *m*/*z* = 339.1964 that appears to lose successive numbers of –CH_2 groups). Right: the positive mode has *m*/*z* peaks corresponding to [$DPG_{dicarbamate}$+H^+] (*m*/*z* = 415.2322) and its various fragmentation products.

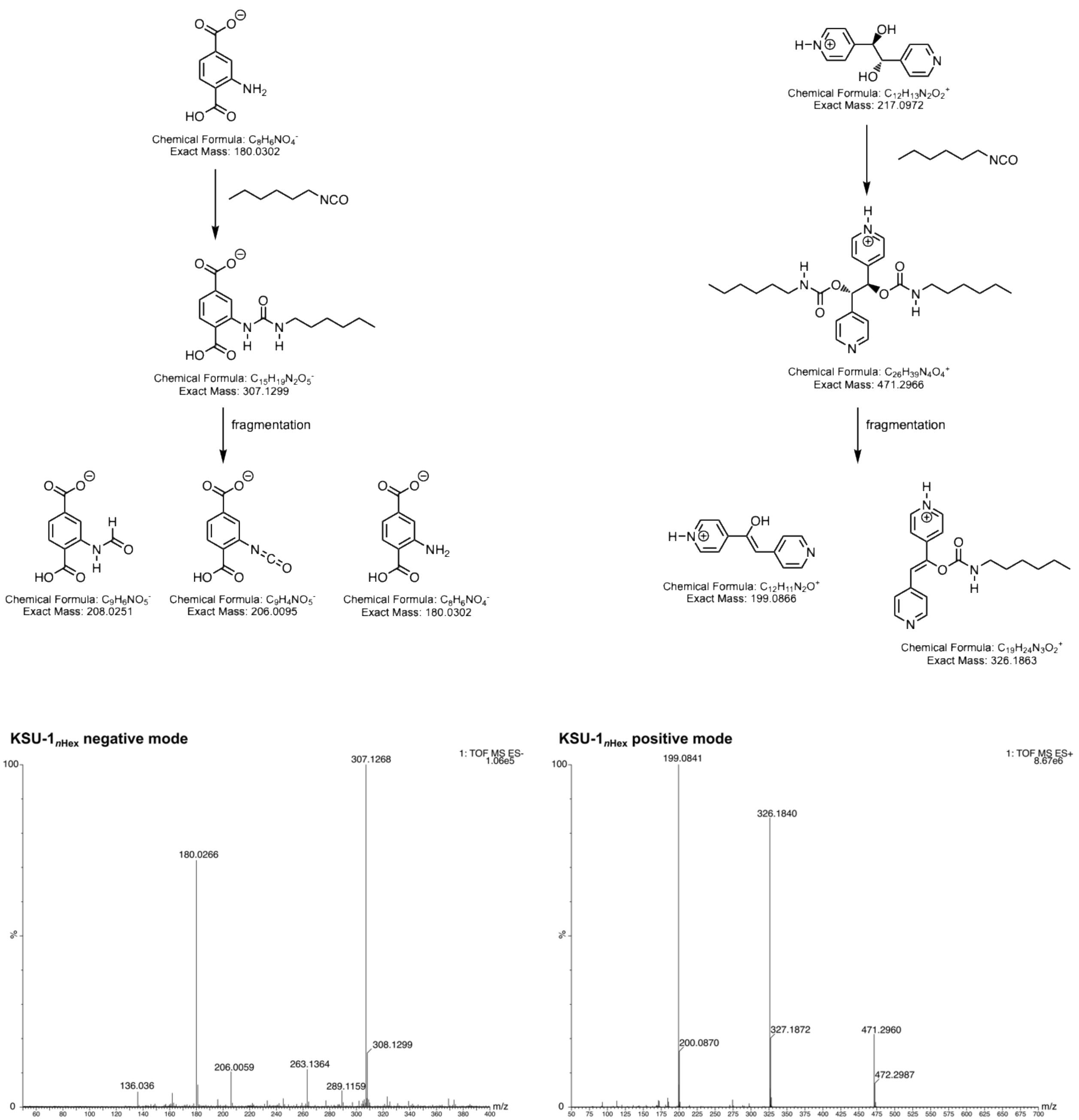

Figure S6: HRMS of KSU-1 after reacting with *n*-hexyl isocyanate to form **KSU-1$_{nHex}$**. Left: the negative mode indicates the presence of the BDC urea product (*m*/*z* = 307.1268), and the [BDC-NH_2-H^+] starting material peak could also be a fragmentation product. Right: the positive mode has *m*/*z* peaks corresponding to [$DPG_{dicarbamate}$+H^+] (*m*/*z* = 471.2960) and its various fragmentation products.

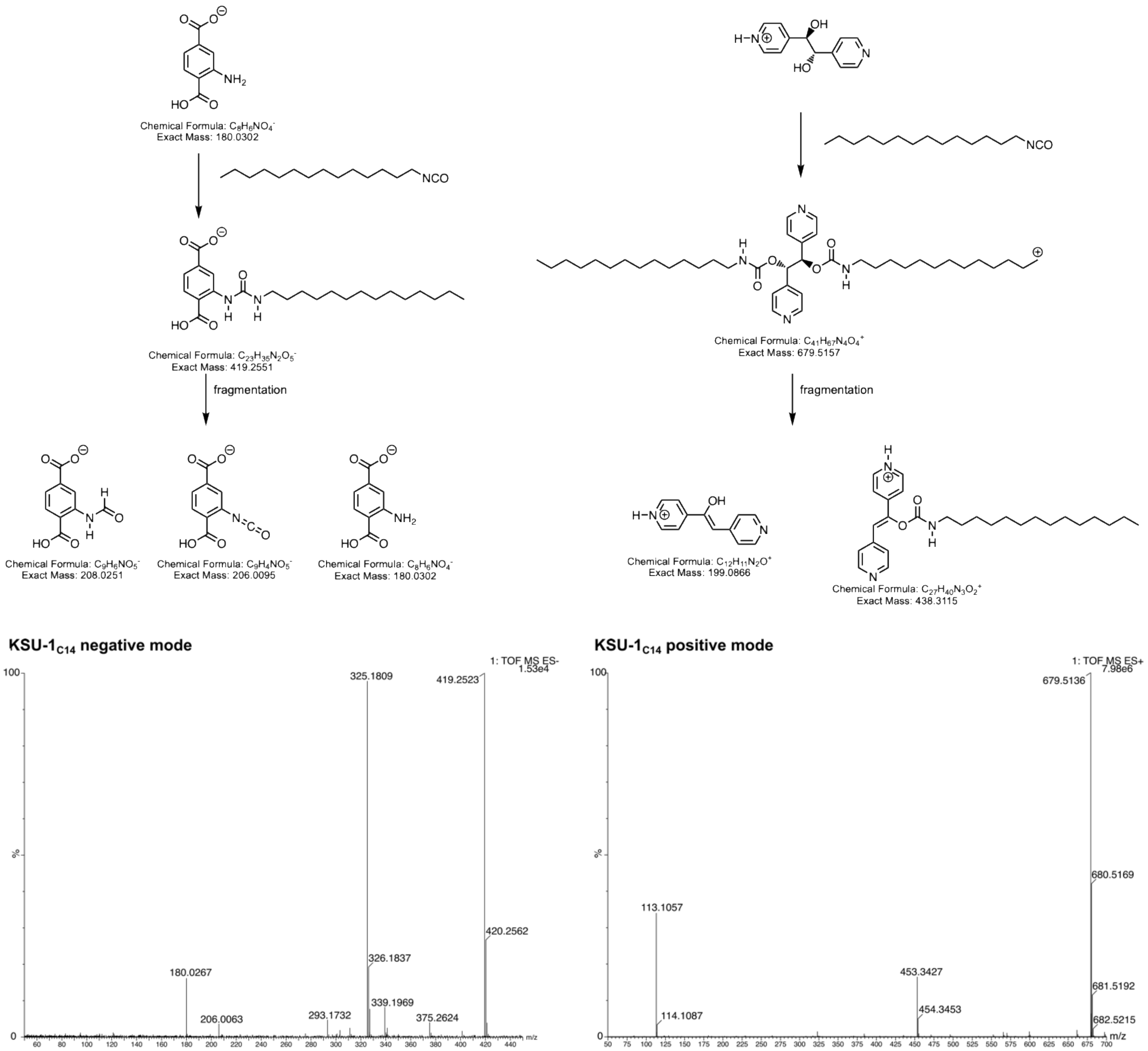

Figure S7: HRMS of KSU-1 after reacting with tetradecyl isocyanate to form **KSU-1$_{C14}$**. Left: the negative mode indicates the presence of the BDC urea product (*m*/*z* = 419.2523), and the [BDC-NH_2-H^+] starting material peak could also be a fragmentation product. Right: the positive mode has *m*/*z* peaks corresponding to [DPG$_{dicarbamate}$-CH_3^-] (*m*/*z* = 679.5136).

POWDER X-RAY DIFFRACTION (PXRD)

Powder diffraction was recorded on a Bruker AXS D8 Advance Phaser diffractometer (Bruker AXS, Karlsruhe, Germany) with Cu Kα radiation (λ = 1.5418 Å) over a range of 4° < 2θ < 40° in 0.02° steps with a 0.5 s counting time per step. Samples were collected from the bottom of the reaction vial as a thick suspension in DMF and spread on a Si-Einkristalle plate immediately before PXRD measurements.

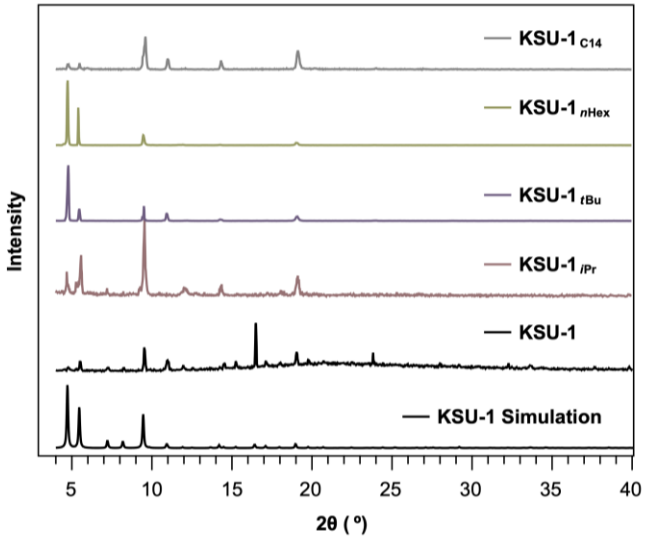

Figure S8. The PXRD patterns of the simulated **KSU-1**, the experimental **KSU-1**, and the **KSU-1** after reacting with the alkyl isocyanates **KSU-1$_{iPr}$** and **KSU-1$_{t\text{-Bu}}$**, **KSU-1$_{n\text{-Hex}}$**, and **KSU-1$_{n\text{-C14}}$**, respectively.

THERMOGRAVIMETRIC ANALYSIS (TGA)

Thermogravimetric analysis was performed on a TGA 8000 (PerkinElmer Inc., Waltham, MA, USA) interfaced with a PC using Pyris software. Samples were heated at a rate of 10 ºC/min under a nitrogen atmosphere. All samples were extensively solvent exchanged with fresh toluene prior to analysis.

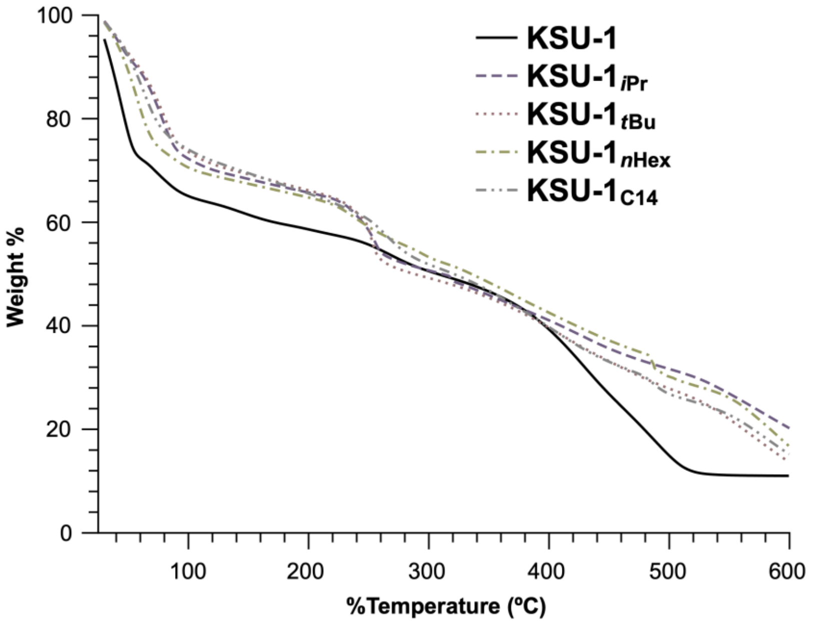

Figure S9. TGA data for as-synthesized **KSU-1, KSU-1$_{iPr}$** and **KSU-1$_{t\text{-Bu}}$**, **KSU-1$_{n\text{-Hex}}$**, and **KSU-1$_{n\text{-C14}}$** exchanged with toluene.
